# Synergistic effects of group-based exercise to reduce loneliness and social isolation and to prevent cognitive decline among older adults with cancer

**DOI:** 10.3389/fragi.2026.1835531

**Published:** 2026-05-25

**Authors:** Heather J. Leach, Jaclyn A. Stephens, Diane K. Ehlers

**Affiliations:** 1 Department of Health and Exercise Science, Colorado State University, Fort Collins, CO, United States; 2 Department of Quantitative Health Sciences, Mayo Clinic, Scottsdale, AZ, United States

**Keywords:** cognition, exercsie training, lonelineness, oncology, social support

## Abstract

The growing population of older cancer survivors faces an elevated risk of accelerated cognitive decline due to the combined effects of aging and cancer-related cognitive impairment. Loneliness and social isolation have also been linked to poorer cognitive outcomes and increased risk of Alzheimer’s Disease and Related Dementias (ADRD). At the same time, regular physical activity and social support interventions may improve cognitive function and reduce risk of cognitive decline. Despite these parallel lines of evidence, few interventions have addressed these modifiable risk factors simultaneously, particularly among older cancer survivors. In this perspective, we propose that group-based exercise may provide synergistic benefits for cognitive health by addressing both physical activity and social engagement. We present a conceptual model in which participation in group exercise promotes exercise-induced neurobiological adaptations that support cognitive function while simultaneously enhancing social connectedness. These processes may reduce loneliness and social isolation, promoting cognitive engagement. We also highlight key considerations for future research, including the potential for virtual or remotely delivered group exercise to increase accessibility, the importance of group dynamics in fostering social support, and the need to incorporate validated measures of loneliness, social isolation, and cognitive function in intervention studies.

## Introduction

In healthy older adults, some degree of age-related cognitive decline is typical (Gonzales et al., 2022). For example, healthy older adults tend to have a working memory capacity that is about 25% smaller than that of healthy younger adults (Schneider-Garces et al., 2010). Some degree of age-related cognitive decline is expected and is gradual and slow, occurring over numerous decades, starting in the 20s and 30s (Salthouse, 2009). In contrast, rapid or accelerated cognitive decline in older adulthood is atypical and can be associated with chronic conditions, like cancer (Wang et al., 2021; Kerstens et al., 2023), or indicative of neurodegenerative disease, like Alzheimer’s Disease and Related Dementias (ADRD) (Gonzales et al., 2022). In older adulthood, preventing accelerated cognitive decline through myriad approaches (e.g., exercise, social engagement) can help reduce risk of ADRD (Schultz et al., 2015). This is essential, as ADRD is a significant public health problem associated with growing healthcare costs (Nandi et al., 2024), reduced independence and quality of life, and increased caregiver burden (Tahami Monfared et al., 2024).

## Older adults with cancer exhibit poorer cognitive function than non-cancer age matched controls

Advances in diagnosis and treatment(s) in oncology have improved survival rates, equating to longer lifespans and a growing number of individuals living with and beyond cancer (i.e., cancer survivors) into older age (Bluethmann et al., 2016). In addition to age-related declines in cognitive function, cancer treatments can induce cognitive deficits, and cancer-associated cognitive decline (CACD) is frequently reported among cancer survivors (Lange et al., 2019). CACD shares many of the same biological pathways as non-cancer related cognitive aging, but these processes may be accelerated in cancer survivors (Mandelblatt et al., 2013; Ahles and Root, 2018). A 2018 review of cancer-related cognitive outcomes found that memory, processing speed, attention and executive function are the most impaired domains (Mandelblatt et al., 2018).

Although the direct link between cancer and ADRD remains inconclusive (i.e., some studies suggest an inverse relationship between cancer and ADRD (Lee et al., 2018), whereas other studies have found that cancer incidence and (ADRD) incidence were strongly correlated (You et al., 2026; Chu et al., 2025), accelerated cognitive decline due to CACD, even in the absence of ADRD, can affect participation in meaningful daily activities, like gainful employment, driving, etc., negatively impacting healthy aging and quality of life.

Physical Activity/Exercise is associated with better cognitive function and reduced risk of ADRD in older adults, including those living with and beyond cancer.

Substantial evidence supports the neurocognitive benefits of regular physical activity and exercise among healthy older adults, and more recent literature supports physical activity for the prevention and management of ADRD and CACD. Data on exercise and CACD, while promising, have generally been focused on the adult cancer population broadly (Sturgeon et al., 2023; Hung et al., 2025; Cam et al., 2020), with few studies focused on exercise’s neurocognitive impacts in *older* cancer survivors specifically. In a recent observational study by Tometich and colleagues (2023), older breast cancer survivors with higher self-reported physical activity exhibited better performance on attention, processing speed, and executive function tasks and reported better perceived cognition at 12–24 months after systemic therapy (Tometich et al., 2023). Likewise, Hanson and colleagues (2020) reported lower cognitive performance among older, compared with younger, breast cancer survivors, but observed positive relationships between self-reported and device-measured physical activity and executive function task performance regardless of age (Hanson et al., 2020). Importantly, surveillance data indicate that only 12% of older adult cancer survivors meet physical activity guidelines for both strength and aerobic activities, with 43% reporting no physical activity at all (Arem et al., 2020). As older adults comprise almost 80% of the cancer survivor population (Wagle et al., 2025) and evidence increasingly indicates greater risk of cognitive decline amid the interaction between cancer and aging (Magnuson et al., 2021), targeted efforts to implement physical activity and exercise to address CACD in older cancer survivors are needed.

Loneliness and social isolation are also linked to cognitive decline and may increase risk of ADRD in older adults.

Social isolation is defined as the objective lack of relationships and loneliness as the perception of lacking social connection (Ohiokpehai et al., 2025). It is estimated that one-quarter of older adults are socially isolated, and one-third are lonely (Cudjoe et al., 2020). Social connectedness is important for maintaining cognitive health and function, particularly among older adults, and social isolation and loneliness among older adults has been linked to increased cognitive decline and ADRD risk. A recent scoping review found that loneliness and social isolation during COVID-19 was correlated with poorer cognitive function in older, community dwelling adults (Kassam and McMillan, 2023), and an analysis of the National Health and Aging Trends Study (a longitudinal and nationally representative cohort of older adults in the U.S.), found that being socially isolated was associated with a 28% higher risk of incident ADRD, even after adjusting for demographic and health factors (Huang et al., 2023). Finally, another study among older adults from a rural region in Southwest Pennsylvania found that social isolation and loneliness were positively associated with cognitive impairment (Fang et al., 2023). A recent review of systematic reviews and meta-analyses found evidence for the inverse relationship; social connections (specifically, social engagement and social activities) were associated with a lower risk of cognitive decline (Joshi et al., 2024). Despite evidence of loneliness and social isolation among cancer survivors (Wang et al., 2024), few studies have examined the relationship between loneliness/social isolation and cognition or ADRD risk specific to older cancer survivors (Koevoets et al., 2022).

Interventions to increase physical activity and reduce loneliness and social isolation to prevent accelerated cognitive decline and potentially reduce ADRD risk in older cancer survivors.

Low levels of physical activity can place already vulnerable older cancer survivors at an even greater risk for loneliness, social isolation, accelerated cognitive decline and ADRD. Fortunately, these factors are modifiable and suggest a need for interventions to mitigate negative outcomes Exercise interventions in older cancer survivors to improve cognitive function.

To date, meta-analyses and randomized controlled trials examining the effects of exercise on cognitive function in cancer populations have not focused on older adults, and samples have been largely skewed toward younger survivors <65 years-old (Koevoets et al., 2022; Brunet et al., 2025), with few exceptions. Fang and colleagues (2020) reported moderate effects of exercise compared to control on self-reported cognitive function across studies in men diagnosed with prostate cancer (median age 67.7 years) (Fang et al., 2020). In a proof of concept, randomized controlled trial, Page and colleagues (2024) observed improvements in functional brain organization (measured via functional magnetic resonance imaging) among post-menopausal breast cancer survivors (mean age 65.9 years) randomized to a 12-week aerobic exercise program (Page et al., 2024). Despite these emerging data, notable limitations in these studies include reliance on self-reported cognition from a quality-of-life scale in the former and a very small sample size in the latter study. Social support interventions in older cancer survivors to reduce loneliness/social isolation.

There is a relatively robust body of literature examining social support interventions among older adults *without* cancer. A 2022 systematic review and meta-analysis found 70 RCT’s to reduce loneliness and/or social isolation in adults >65 years old (Hoang et al., 2022). Intervention types varied widely; eight studies included exercise and showed a small effect, and the largest effects on social isolation or loneliness were attributed to animal therapy and videoconferencing. More recently, a 2024 systematic review examined the cognitive effects of interventions to reduce social isolation and loneliness in older adults (Baptista et al., 2024). This review yielded nine studies, with six showing improvements in cognitive function post-intervention, and of these, five utilized remote or technology-based delivery modalities. Only one study included exercise as part of one of its intervention arms.

The literature exploring social support interventions among cancer survivors is sparser. A 2021 systematic review and meta-analysis of interventions to address loneliness among cancer survivors revealed a limited number of interventions (*n* = 8) (McElfresh et al., 2021). Promisingly though, findings from these few studies suggest that loneliness is modifiable among cancer survivors, but few interventions have been tested for effectiveness. Importantly, at the time of this review, none of these interventions included physical activity or exercise; none were tailored for older adult survivors; and none examined markers of cognitive function as an outcome. Most recently, a 2026 pilot RCT examined home-based exercise plus two forms of social support (peer support with or without professional support) among older adults (aged≥65 years) living with or beyond a cancer diagnosis, and found that the intervention was feasible, and those in the peer + virtual professional support demonstrated improved exercise-related social support (Smith-Turchyn et al., 2026). Another 2025 study conducted a secondary data analysis of an exercise trial to explore the potential for virtual, group-based exercise to reduce loneliness among prostate cancer survivors (mean age 71 years) (Winters-Stone et al., 2025).

In general, more research is needed to examine the effects of interventions to reduce social isolation and loneliness among older cancer survivors and determine whether these interventions can help prevent or ameliorate accelerated declines in cognitive function.

## Synergistic effects of group-based exercise to reduce loneliness and social isolation

**FIGURE 1:**
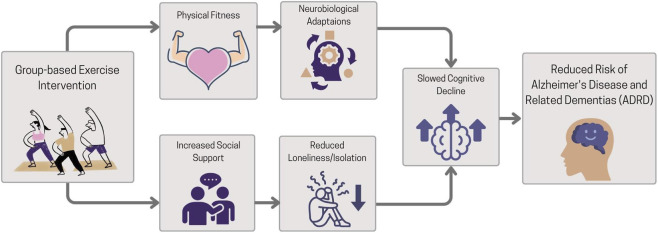
Conceptual model for group based exercise to reduce loneliness/social isolation and prevent accelerated cognitive decline.

In this section we present a conceptual model (Figure 1) to depict hypothesized pathways through which a group-based exercise intervention may help prevent accelerated cognitive decline among older adults with cancer and potentially reduce subsequent ADRD risk. We propose that participation in group exercise will (a) elicit the known exercise-induced neurobiological adaptations associated with improved cognition and (b) enhance social connectedness and perceived social support, leading to reductions in loneliness and social isolation. Structured, repeated social interaction combined with exercise creates opportunities for relationship building, shared goals, and accountability. Participation in group exercise fosters emotional, informational, and companionship support from peers and instructors, resulting in enhanced social connectedness and support, which can buffer perceived isolation and loneliness (Sebastiao and Mirda, 2021; Li et al., 2024). Reduced isolation/loneliness is associated with lower chronic stress (Ehlers et al., 2017), improved mood, and greater cognitive engagement, supporting domains such as executive function, memory, and processing speed. Beyond that, the understanding of the mechanisms underlying the relationship between social isolation/loneliness and cognitive decline is limited (Lam et al., 2021; Friedler et al., 2015).

In summary, exercise-induced neurobiological adaptations combined with reductions in loneliness and/or social isolation are hypothesized to work synergistically to positively impact cognitive function, thereby contributing to a reduced risk of ADRD over time.

### Intervention considerations

When conceptualizing interventions that combine physical activity/exercise and social support to prevent or reduce accelerated cognitive decline among older cancer survivors, several important elements should be considered, including remote delivery, source(s) of social support, and outcome measures.

### Remote delivery

Strategies to enhance social connectedness in face-to-face or “in-person” group-exercise settings are well-established (Estabrooks et al., 2012; Burke et al., 2006), including a systematic review of group-based interventions for cancer survivors (Leach et al., 2019a). However, one of the greatest barriers to participation in physical activity/exercise trials in cancer survivorship is transport and distance from the intervention site (Reynolds et al., 2023). Subsequently, older adults living in rural or remote areas exhibit a higher prevalence of social isolation/loneliness (Hussain et al., 2023), likely due to structural barriers that reduce opportunities for social connection (Henning-Smith, 2020). Thus, advances in virtual/remote options can increase opportunities and access to group-based exercise interventions and programs for older cancer survivors. One previous study demonstrated that access to technology alone was associated with a lower risk for social isolation among community-dwelling older adults (Umoh et al., 2023), and the rapid uptake of videoconference technology among older adults during the COVID-19 pandemic (Choi et al., 2022) uncovered an important opportunity for remotely delivered interventions to reduce social isolation and loneliness (Welch et al., 2023). The feasibility and acceptability of videoconference technology to deliver group-based exercise to cancer survivors has been established (Smith et al., 2025; Leac et al., 2023; Crisafio et al., 2024), and to our knowledge, only one study has explored the outcome of loneliness or social isolation (Winters-Stone et al., 2025), with another large trial currently ongoing (NCT07290309). Therefore, if there is a need to increase reach and/or accessibility to a group-based exercise intervention or program, virtual/remote delivery (i.e., videoconferencing) should be considered as a viable option.

### Sources of support

Physical activity and/or exercise contexts can facilitate social relationships for cancer survivors, but the degree of closeness in those relationships is highly variable (McDonough et al., 2021), and the optimal source of social support is unclear (McDonough et al., 2019).

The exercise group in the context of a physical activity intervention or program can be a robust source of social support. The inclusion of strategies based in group dynamics and the composition of the group can influence the level or intensity of connection, and subsequently, the extent to which this may reduce loneliness or social isolation. Group dynamics strategies target the group’s environment, structure, and processes, which are used to enhance cohesion among participants in an exercise group (Spink and Carron, 1994; Carron et al., 1996). Physical activity interventions that include group dynamics principles to increase cohesiveness have been shown to be most effective for increasing physical activity adherence (Leach et al., 2019a; Emslie et al., 2007; Pinto et al., 2015) and can offer a new network of social support for (Beauchamp et al., 2015). The composition of exercise groups may also play an important role in the degree of social connection that is facilitated. For example, groups composed exclusively of cancer survivors may provide an important opportunity to be surrounded by others with a shared experience (Leach et al., 2019a; Leach et al., 2019b; Emslie et al., 2007). Previous studies have examined the effect of the composition of these groups on PA adherence (e.g., similar age and/or sex, inclusion of a peer mentor) (Pinto et al., 2015; Beauchamp et al., 2015), but how the composition of the exercise group impacts loneliness/social isolation is not well established.

### Outcomes

As depicted in Figure 1, a group-based exercise intervention for older adult cancer survivors is expected to decrease loneliness and social isolation and elicit neurobiological adaptations which positively impact cognitive function Together, these changes are expected to, over time, slow cognitive impairment and potentially reduce risk of ADRD in older adult cancer survivors. Subsequently, in order to expound effects of these interventions, there is a need to include the key outcome measures of social isolation and/or loneliness, and cognitive function.

At present, a consensus does not exist for a measure of social isolation for older adults (Pomeroy et al., 2023), but a recent review does provide five recommended evidence-based measures (Gardam et al., 2023), which can help guide researchers in selecting a measure to assess social isolation and/or examine intervention effectiveness on this outcome. Similarly, a review of loneliness measures among older adults found no unanimity on one measure, but that reliability and validity exist across multiple available measures (Newmyer et al., 2021). In terms of frequency and timing of assessing loneliness and/or social isolation, measures should be implemented at pre- and post-intervention and, if possible, as a screening or inclusion criterion, as well as mid- or part way through the intervention so that adaptations to enhance social connection or engagement can be made.

To evaluate cognitive function, cognitive screening tools, like the mini-mental status exam (MMSE) (Folstein et al., 1975) or Montreal Cognitive Assessment (MoCA) (Nasreddine et al., 2005), that can track cognitive status over time and screen for precursors of ADRD, like mild cognitive impairment (MCI), are important. These cognitive screening tools could be complemented with secondary outcome measures of cognitive function, including longer neurocognitive assessments, like the Automated Neuropsychological Assessment Metrics (ANAM) (Reeves et al., 1995), and functional cognitive assessments, like the Weekly Calendar Planning Activity (Toglia and Berg, 2013), which has been used to detect post-intervention improvements in the everyday cognitive performance of older adults (Stephens and Berryhill, 2016). Additional secondary outcome measures may include those that measure participation in meaningful roles and activities (e.g., Activity Card Sort) (Boone et al., 2022), as reduced loneliness and better cognitive performance can improve participation, which is also associated with reduce risk of ADRD (Duffner et al., 2022). Like loneliness and social isolation, atypical cognitive status should be measured during screening using tools like MMSE or MOCA. Because exercise is a complex behavior that relies upon executive function and self-regulatory skills, understanding survivors’ baseline cognitive status is critical to identifying appropriate candidates for an exercise intervention and providing individualized strategies that help individuals adhere to regular exercise (McAuley et al., 2011). Neurocognitive batteries assessing cognitive function in domains impacted by aging and cancer should be administered at pre- and post-intervention. Longer-term follow-up (e.g., 6 months up to 5 years post-intervention) assessments including neurocognitive assessments and cognitive screening, may also be considered to understand the maintenance effects of reduced loneliness and increased exercise on cognitive function and impairment over time.

## Summary

Older cancer survivors are at heightened risk for accelerated cognitive decline, and although physical activity and social support independently show promise for preserving cognitive health, previous interventions have rarely targeted these modifiable pathways in an integrated manner. We propose that by combining exercise-induced adaptations with enhanced social connectedness, such interventions may reduce loneliness, increase cognitive engagement, and ultimately mitigate risk for cognitive decline. Future research should prioritize scalable delivery models, including remote group-based formats, consider integrating group dynamics principles, and incorporate rigorous, validated assessments of loneliness and/or social isolation and cognitive outcomes.

## Data Availability

The original contributions presented in the study are included in the article/supplementary material, further inquiries can be directed to the corresponding author.
